# Bradykinin reduces wound healing in human umbilical vein endothelial cells via downregulation of vascular endothelial growth factor A

**DOI:** 10.1186/s12950-026-00485-x

**Published:** 2026-01-10

**Authors:** Nevena Dimitrova, Angelina Gierke, Raphael Möhrle, Julia Nemeth, Cornelia Brunner, Thomas K. Hoffmann, Jens Greve, Janina Hahn, Robin Lochbaum

**Affiliations:** 1 Department of Otorhinolaryngology, Head and Neck Surgery, Ulm University Medical Center, Frauensteige 12, 89075 Ulm, Germany; 2 Department of General Physiology, Ulm University, Ulm, Germany; 3 Core Facility Immune Monitoring, Ulm University Medical Faculty, Ulm, Germany

## Abstract

**Introduction:**

Hereditary angioedema (HAE) is characterized by acute swelling attacks triggered by abnormally elevated levels of bradykinin. Despite persistently high bradykinin levels, patients experience only intermittent swelling episodes. Many HAE patients, however, report triggers such as trauma preceding angioedema attacks. This suggests the involvement of additional factors, such as mechanical damage to the endothelium. Bradykinin-mediated impairment of wound healing may contribute to swelling development—a concept known as the “second hit” hypothesis. Vascular endothelial growth factors (VEGF) and their receptors play a critical role in endothelial wound healing and have also been implicated in bradykinin-mediated angioedema. This study investigates the influence of bradykinin on endothelial wound healing, with a particular focus on VEGF.

**Materials and methods:**

Human umbilical vein endothelial cells were incubated with bradykinin and VEGF. Gene and protein expression were analyzed by real-time polymerase chain reaction, western blotting, and immunocytochemistry. Barrier function was assessed by measuring transendothelial electrical resistance, as well as apparent and water permeability. Proliferation rates were determined using resazurin assays and real-time cell analysis. Cell migration was assessed using invasion and migration assays, and the combined effects were evaluated using scratch assays.

**Results:**

Bradykinin treatment led to reduced expression of the VEGFA isoform and its receptor VEGFR-2. VEGFA alone had no effect on bradykinin-induced barrier disruption. However, bradykinin significantly decreased endothelial proliferation and migration, resulting in impaired wound healing. This effect was counteracted by the addition of VEGFA.

**Conclusions:**

VEGFA and its receptor VEGFR-2 are key regulators of endothelial wound healing. Bradykinin impairs wound healing by reducing proliferation and migration, likely through downregulation of VEGFA and VEGFR-2. These findings support the hypothesis that bradykinin-mediated impairment of wound healing may contribute to the episodic nature of swelling attacks in patients with bradykinin-mediated angioedema, in line with the second hit hypothesis.

**Supplementary Information:**

The online version contains supplementary material available at 10.1186/s12950-026-00485-x.

## Introduction

The vasoactive nonapeptide bradykinin is one of the most important mediators of the kallikrein-kinin system [1]. Activation of the bradykinin-generating cascade is closely associated with inflammatory processes. Two kinin receptor subtypes have been identified and characterized: bradykinin B1 receptor and bradykinin B2 receptor, both belonging to the family of G protein-coupled receptors [2]. Bradykinin exerts a wide range of physiological effects, among which inflammation, pain, vasodilation, and increased vascular permeability in the human endothelium are the most prominent. However, the precise molecular mechanisms underlying the increase in vascular permeability remain incompletely understood. In human blood vessels, the endothelial barrier and its permeability are maintained by cell-to-cell junctions [3]. These junctions not only regulate the exchange of fluids, solutes, and cells but also play critical roles in processes such as cell proliferation, migration, and adhesion.

An example of uncontrolled bradykinin-induced endothelial permeability is the rare genetic disorder hereditary angioedema (HAE). C1 esterase inhibitor (C1-INH) deficiency-dependent HAE is caused by mutations in the SERPING1 gene, which encodes C1INH [4]. A deficiency of C1INH leads to elevated bradykinin concentrations in the blood of affected individuals [5]. Clinically, HAE is characterized by recurrent, acute swelling episodes, known as angioedema, which are often painful, disabling, and potentially life-threatening, particularly when involving the head and neck region [6]. Interestingly, although bradykinin levels are persistently elevated in HAE patients, swelling episodes occur episodically rather than continuously. Several trigger factors are known to increase the likelihood of an angioedema attack, and most patients are able to identify such triggers. The most common include mental stress, hormonal changes, infections, and mechanical stimuli [7]. These observations suggest the involvement of additional molecular factors that may promote the onset of an attack, potentially through mechanical damage to the endothelium or infection—a concept referred to as the “second hit” hypothesis [8–10]. Minor (micro)injuries may lead to endothelial damage, which under normal physiological conditions is rapidly repaired, preventing significant leakage of water and solutes. However, due to its pro-inflammatory properties, bradykinin may impair endothelial wound healing, thereby promoting edema formation. The second hit hypothesis is further supported by reports from patients, who often recall minor trauma or mechanical irritation preceding an attack [11].

One modulator of bradykinin-mediated barrier disruption is vascular endothelial growth factor (VEGF). The term VEGF refers to a family of proteins, including VEGFA, VEGFB, VEGFC, VEGFD, and placental growth factor (PLGF). As the name suggests, VEGF is a key factor that promotes endothelial cell proliferation [12]. Members of the VEGF family exert their effects by binding to specific tyrosine kinase receptors, known as VEGF receptors (VEGFRs). The most important of these are VEGFR-1 (FLT1 = fms-related tyrosine kinase 1), VEGFR-2 (KDR/FLK1 = kinase insert domain receptor/fetal liver kinase 1), and VEGFR-3 (FLT4 = fms-related tyrosine kinase 4). VEGFA primarily binds to VEGFR-1 and VEGFR-2, with the latter being responsible for initiating the majority of VEGF-mediated signaling pathways [13]. Altered VEGF expression is associated with the pathogenesis of various diseases, including age-related macular degeneration, cancer, ischemic heart disease, rheumatoid diseases, chronic obstructive pulmonary disease, asthma, and urticaria [14]. Previous studies have reported elevated levels of VEGFA and VEGFC in patients with ACE inhibitor-induced angioedema [15]. Bradykinin is known to influence the expression of VEGF, particularly the VEGFA isoform, although there are also data indicating effects on VEGFC [16]. However, the literature presents conflicting findings, with some studies reporting an upregulation of VEGF expression, while others observe the opposite effect [17]. The effect of VEGF on endothelial barrier function also appears to be context dependent. In some types of endothelia, VEGF is thought to disrupt the barrier [18], whereas in others it seems to have no significant effect [19]. In this context, VEGF emerges as a relevant factor in the study of bradykinin-mediated endothelial barrier dysfunction. To date, the precise molecular interaction between bradykinin and VEGF remains unclear, as does the role of VEGF in angioedema at the molecular level. Therefore, the present study aimed to analyze the role of VEGF in bradykinin-induced impairment of endothelial cell proliferation in angioedema. This may contribute to a deeper understanding of the pathogenesis of acute bradykinin-mediated angioedema.

## Materials and methods

### Cell culture

The experiments were performed using human umbilical vein endothelial cells (HUVECs), a widely accepted endothelial cell model. The cells were cultured in endothelial cell growth medium supplemented with fetal calf serum (FCS, 0.02 ml/ml), epidermal growth factor (EGF, 5 ng/ml), basic fibroblast growth factor (FGF, 10 ng/ml), insulin-like growth factor (IGF, 20 ng/ml), vascular endothelial growth factor (VEGF, 0.5 ng/ml), ascorbic acid (1 µg/ml), hydrocortisone (0.2 µg/ml), heparin (22.5 µg/ml), and 2% penicillin/streptomycin (all from *PromoCell GmbH*,* Heidelberg*,* Germany*). Cells up to passage 4 were used for all experiments.

After reaching confluency, cells were seeded on semipermeable filter inserts with a pore size of 0.4 μm (*Sarstedt AG & Co. KG*,* Nümbrecht*,* Germany*) in 24-well cell culture plates. Prior to seeding, the inserts were coated overnight with a collagen solution consisting of 5 mg collagen I (rat tail) per 100 ml of 0.02 M acetic acid solution (both from *Thermo Fisher Scientific*,* Karlsruhe*,* Germany*). The medium was changed on the second day after cultivation on the filters. Unless specified otherwise, all experiments were conducted on the third day.

The following modulators were added for 4 h at the indicated concentrations: bradykinin (100 µmol/l, *Sigma-Aldrich Chemie GmbH*,* Steinheim*,* Germany*), icatibant (10 nmol/l, *Takeda GmbH*,* Berlin*,* Germany*), VEGF-165 Human Recombinant Protein (100 ng/ml, *Thermo Fisher Scientific*,* Karlsruhe*,* Germany*), and sunitinib (100 nmol/l, *Cell Guidance Systems Ltd.*,* Cambridge*,* UK*).

### Real time - polymerase chain reaction (RT-PCR)

HUVECs were seeded at a density of 20,000 cells/well on semipermeable filter inserts with a pore size of 0.4 μm (*Sarstedt AG & Co. KG*,* Nümbrecht*,* Germany*) in 24-well cell culture plates (*Thermo Fisher Scientific*,* Karlsruhe*,* Germany*) and cultured for four days until confluence. VEGF, icatibant and bradykinin were then added at the concentrations listed above. After four hours, RNA was extracted using the RNeasy Mini Kit (*QIAGEN GmbH*,* Hilden*,* Germany*) according to the manufacturer’s protocol. Meanwhile, DNA was enzymatically digested using the RNase-Free DNase Kit (*QIAGEN GmbH*,* Hilden*,* Germany*), also following the manufacturer’s instructions. cDNA was synthesized using the QuantiTect Reverse Transcription Kit (*QIAGEN GmbH*,* Hilden*,* Germany*) in a peqSTAR 96X Universal Gradient Thermocycler (*VWR International GmbH*,* Darmstadt*,* Germany*) at 42 °C for 15 min and 95 °C for 3 min. Primer sequences for RT-PCR were selected from the NCBI database. The following primer pairs were used: GAPDH (forward: agccacatcgctcagacac; reverse: gcccaatacgaccaaatcc), VEGFA (forward: aggccagcacataggagagatg; reverse: accgggatttcttgcgctttc), VEGFB (forward: gggggagatgtccctggaagaa, reverse: cccggaacagaacggggct), VEGFC (forward: aacagagaacaggccaacctca; reverse: tcccgtggcatgcattgagt), VEGFD (forward: gaacaccagcacctcgtaca; reverse: ggctgttggcaagcacttaca), PGF (forward: agccacatcgctcagacac; reverse: gcccaatacgaccaaatcc), VEGFR-1 (forward: aggccgtgtcatcgtttcca; reverse: ggggtgccagaaccacttga), VEGFR-2 (forward: acaagaccaaaggggcacga; reverse: ccaagcgccgtttcagatcc) and VEGFR-3 (forward: tgtggctctgcctgggactc; reverse: cctgcaggagatggacaggc). The corresponding primers were synthesized by *biomers.net GmbH*,* Ulm*,* Germany*. Glyceraldehyde-3-phosphate dehydrogenase (GAPDH) was used as a housekeeping gene. RT-PCR was performed according to the protocol of the QuantiNova™ SYBR^®^ Green PCR Kit (*QIAGEN GmbH*,* Hilden*,* Germany*) in a LightCycler^®^ 480 System (*Roche Deutschland Holding GmbH*,* Grenzach-Wyhlen*,* Germany*) according to the following program: 95 °C for 2 min followed by 40 cycles of 95 °C for 5 s and 60 °C for 10 s.

### Western blot (WB)

HUVECs were seeded in 25 cm² cell culture flasks. VEGF and bradykinin were added at the appropriate concentrations, and after four hours, the cells were resuspended. After centrifugation (4 °C, 250 × g for 10 min), the supernatant was discarded, and the pellet was washed with 1 ml Dulbecco’s Phosphate-Buffered Saline (DPBS, *PAN-Biotech*,* Aidenbach*,* Germany*). A second centrifugation (4 °C, 250 × g for 10 min) followed; the supernatant was discarded, and the cell pellet was resuspended in lysis buffer (25 mM Trizma base, 150 mM sodium chloride, 1% Triton X-100, 0.1% ultrapure SDS, 1% sodium deoxycholate; all from *Sigma-Aldrich Chemie GmbH*,* Steinheim*,* Germany*).

The mixture was incubated for 30 min on ice, centrifuged (4 °C, 1500 × g for 10 min), and the supernatant was transferred to a new reaction tube. Protein quantification was performed using the Pierce™ BCA Protein Assay Kit according to the manufacturer’s protocol (*Thermo Fisher Scientific*,* Karlsruhe*,* Germany*).

Protein samples (20 µg in radioimmunoprecipitation assay buffer) were mixed with Laemmli buffer containing 2-mercaptoethanol (10:1, *Bio-Rad Laboratories GmbH*,* Feldkirchen*,* Germany*) and denatured at 95 °C for 5 min. Proteins were separated by SDS-PAGE using standard stacking and separating gels and transferred onto membranes with the Trans-Blot^®^ Turbo™ Transfer System (*Bio-Rad Laboratories GmbH*,* Feldkirchen*,* Germany*). Membranes were washed three times with TBS-T (*Sigma-Aldrich Chemie GmbH*,* Steinheim*,* Germany)* and blocked in 5% BSA for 1 h at room temperature. Protein ladders (Precision Plus Protein™ Dual Color Standards, *Sigma-Aldrich Chemie GmbH*,* Steinheim*,* Germany*) were used for size reference.

The primary antibody (VEGF Monoclonal Antibody MA1-16629, *Thermo Fisher Scientific*,* Karlsruhe*,* Germany*) was diluted 1:1000 in 5% milk/TBS-T and incubated overnight at 4 °C. The membrane was then washed three times for five minutes in TBS-T. The secondary antibody (anti-mouse IgG antibody produced in goat, M4155, *Sigma-Aldrich Chemie GmbH*,* Steinheim*,* Germany*) was diluted 1:10,000 in 5% BSA/TBS-T and incubated for 1 h at room temperature. The membrane was then washed three times for five minutes in TBS-T and developed using chemiluminescence with the ChemiDoc™ MP Imaging System (*Bio-Rad Laboratories GmbH*,* Feldkirchen*,* Germany*).

### Immunocytochemistry (ICC)

Cells were cultured on a µ-Slide 8-well chamber slide (*ibidi GmbH*,* Gräfelfing*,* Germany*). After reaching confluence, the cells were washed with phosphate-buffered saline containing calcium and magnesium (PBS; *PAN-Biotech GmbH*,* Aidenbach*,* Germany*). Fixation was performed with 4% formaldehyde (*Thermo Fisher Scientific*,* Karlsruhe*,* Germany*) in PBS at room temperature for 10 to 20 min. After three additional washes with PBS, the cells were permeabilized with 0.1% Triton X-100 (*Sigma-Aldrich Chemie GmbH*,* Steinheim*,* Germany*) in PBS for 5 min, followed by two 5-minute incubations in 0.5% Triton X-100 with 2% fetal calf serum (FCS) in PBS. Primary antibodies (VEGF Monoclonal Antibody MA1-16629 and VEGF Receptor 2 Monoclonal Antibody MA5-15556, both from *Thermo Fisher Scientific*,* Karlsruhe*,* Germany*) were diluted 1:100 in PBS containing 2% FCS and applied to the cells for 1 h at room temperature. After three washes with PBS containing 2% FCS, the secondary antibody (Goat anti-mouse IgG Cross-adsorbed Secondary Antibody, Alexa Fluor™ 594, A-11005; *Thermo Fisher Scientific*,* Karlsruhe*,* Germany*) was diluted 1:400 in PBS with 2% FCS. For nuclear staining, Hoechst 33,342 (*Thermo Fisher Scientific*,* Karlsruhe*,* Germany*) was added at a dilution of 1:50,000. Cells were incubated in this solution for 1 h at room temperature. Microscopy was performed using a KEYENCE BZ-9000 fluorescence microscope (*Keyence Deutschland GmbH*,* Neu-Isenburg*,* Germany*) after three washes with PBS containing 2% FCS followed by three washes with PBS alone. Image analysis was carried out using FIJI (based on ImageJ; *National Institutes of Health*,* Bethesda*,* USA*).

### Transendothelial electrical resistance (TEER)

On the third day after plating, the filter inserts were transferred to the cellZscope (*nanoAnalytics GmbH*,* Münster*,* Germany*), and 250 µl of culture medium was added to the apical compartment and 500 µl to the basolateral compartment. Analysis was conducted using the manufacturer’s software.

### Apparent permeability coefficient (P_app_)

P_app_ was assessed using fluorescein isothiocyanate (FITC)-coupled 70 kDa dextrans (*Sigma-Aldrich Chemie GmbH*,* Steinheim*,* Germany*). On the third day, 100 µl isotonic saline was added to the apical compartment and 500 µl of culture medium containing the dextran to the basolateral compartment. After one hour, the solution in the apical compartment was removed. A standard dilution series of known dextran concentrations was prepared. Fluorescence intensity was measured using the Infinite M200 plate reader (*Tecan Group AG*,* Männedorf*,* Switzerland*), and the corresponding dextran concentration was determined from the calibration curve. P_app_ was then calculated using the following equation P_app_ = (C × V_0_)/(∆t × A × ∆C_0_). Where C = marker concentration after incubation time, V_0_ = apical volume of isotonic saline at baseline, ∆t = incubation time, A = filter area, ∆C_0_ = difference in marker concentration between apical and basal compartments at t = 0.

### Determination of transendothelial water flux using D_2_O dilution method

Transendothelial water flux across transwell filter inserts was determined using the D₂O dilution method. This protocol was recently established in our working group and published [20]. Water flux was induced by a hydrostatic pressure gradient (1 cmH₂O, 24 h) applied via apical 0.9% NaCl. After incubation, 25 µl of isotonic D₂O was added apically, mixed, and the total apical volume collected. The H₂O/D₂O ratio was measured using Fourier-transform infrared spectroscopy. Each molecular bond absorbs infrared light at characteristic wavelengths, allowing the specific H₂O and D₂O content to be quantified. Using a dilution series, the H₂O/D₂O ratio was used to calculate the volume of water transported across the endothelium.

### Resazurin viability assay

The non-fluorescent blue dye resazurin is reduced in the mitochondria of viable cells to the fluorescent compound resorufin. The resulting fluorescence intensity correlates directly with the number of viable cells, making this assay suitable for assessing proliferation rates. The Resazurin Assay Kit (*Abcam*,* Cambridge*,* UK*) was used according to the manufacturer’s instructions.

### Scratch assay

HUVECs were grown to confluence. After incubation with the appropriate chemical, a mechanical scratch was introduced using a pipette tip. The combined effects of proliferation and migration as indirect indicators of wound healing were assessed after 6 h using an Olympus CK30 microscope (*Carl Zeiss AG*,* Oberkochen*,* Germany*) at 10× magnification. Image processing and calculation of the remaining wound area were performed according to the original publication [21].

### Real time cell analysis (RTCA)

Real-time cell analysis was conducted using the xCELLigence RTCA system (*Agilent Technologies Germany GmbH & Co. KG*,* Waldbronn*,* Germany*). Cells were cultured in specialized plates equipped with gold electrodes. As cells act as electrical insulators, their attachment to the electrode surface leads to changes in impedance, which is reported as the cell index (CI). RTCA E-Plates were used to assess proliferation. Cells were seeded onto the gold electrodes, and changes in CI were monitored over time. Proliferation was quantified by calculating the slope (gradient) of the CI curve. For wound healing experiments, cells were grown to confluence (indicated by a plateau in CI), followed by a scratch using a pipette tip, ensuring the electrodes were not damaged. The time taken for the CI to return to plateau levels was used as an indicator of wound closure. RTCA CIM (Cell Invasion and Migration) plates were used to study endothelial cell migration, following the manufacturer’s instructions. Cells were seeded in the upper chamber containing Corning Matrigel Basement Membrane Matrix (*Merck KGaA*,* Darmstadt*,* Germany*). In the lower chamber, VEGF was added at a concentration of 100 ng/ml as a chemoattractant. As a control, background migration was measured using medium without VEGF in both chambers. Migration across the porous membrane was detected via impedance changes and displayed as a change in CI.

### Statistical analysis

Prism 5 (*GraphPad*,* San Diego*,* USA*) was used for data analysis. Statistical tests are specified in the text. Differences were considered statistically significant at *p* < 0.05. Significance levels were indicated in the figures as follows: * = *p* < 0.05, ** = *p* < 0.01, *** = *p* < 0.001.

## Results

### Bradykinin significantly downregulates VEGFA expression

**Fig. 1:**
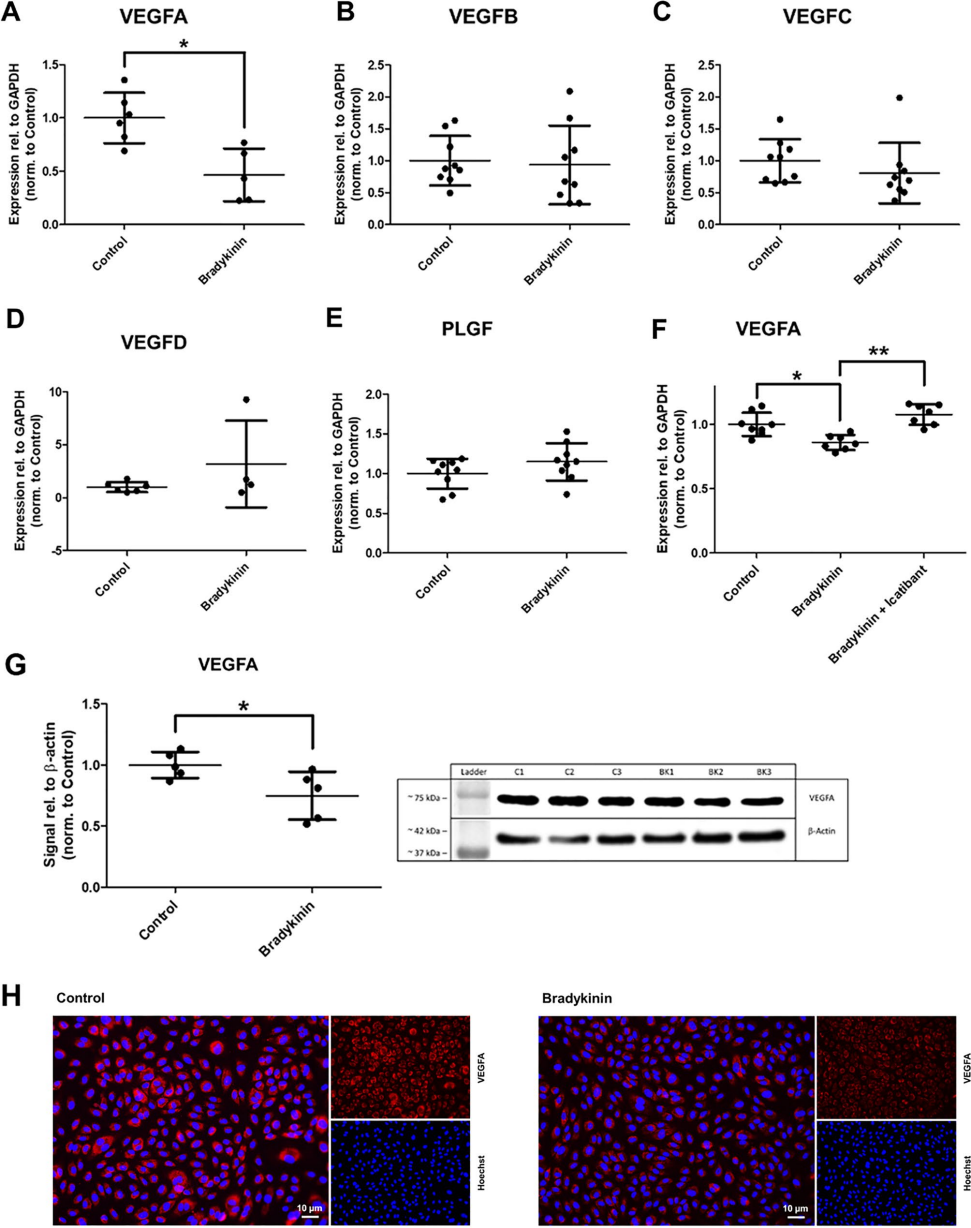
Bradykinin (100 µmol/l, 4 h) downregulates vascular endothelial growth factor (VEGF)A. RT-PCR analysis showing expression of VEGFA (**A**), VEGFB (**B**), VEGFC (**C**), VEGFD (**D**), and placental growth factor (PLGF, **E**). The bradykinin-induced downregulation of VEGFA was reversed by the B2 receptor antagonist icatibant (10 nmol/l, 4 h; **F**). Expression levels were normalized to glyceraldehyde-3-phosphate dehydrogenase (GAPDH). Downregulation of VEGFA was further confirmed by Western blot analysis (**G**; representative blot, normalized to β-actin) and immunocytochemical staining (**H**; VEGFA in red, nuclei in blue). The values are shown as scatter plots and the mean value with standard deviation is given. Each data point represents one filter. Significance was calculated using the two-tailed Mann-Whitney test. (* = *p* < 0.05, ** = *p* < 0.01)

HUVECs were used as a widely accepted endothelial cell model for these investigations. First, the influence of bradykinin on the VEGF signaling pathway was examined (Fig. 1). Treatment of HUVECs with 100 µM bradykinin resulted in a significant downregulation of VEGFA at both the gene and protein expression levels. Addition of bradykinin B2 receptor antagonist icatibant counteracted the effect of bradykinin. ICC showed no relevant change in localization but revealed an overall weaker signal, which was confirmed by WB experiments. However, bradykinin had no effect on the expression levels of VEGFB, VEGFC, VEGFD, or PLGF.

### Bradykinin significantly decreases the expression of VEGFR-2

**Fig. 2:**
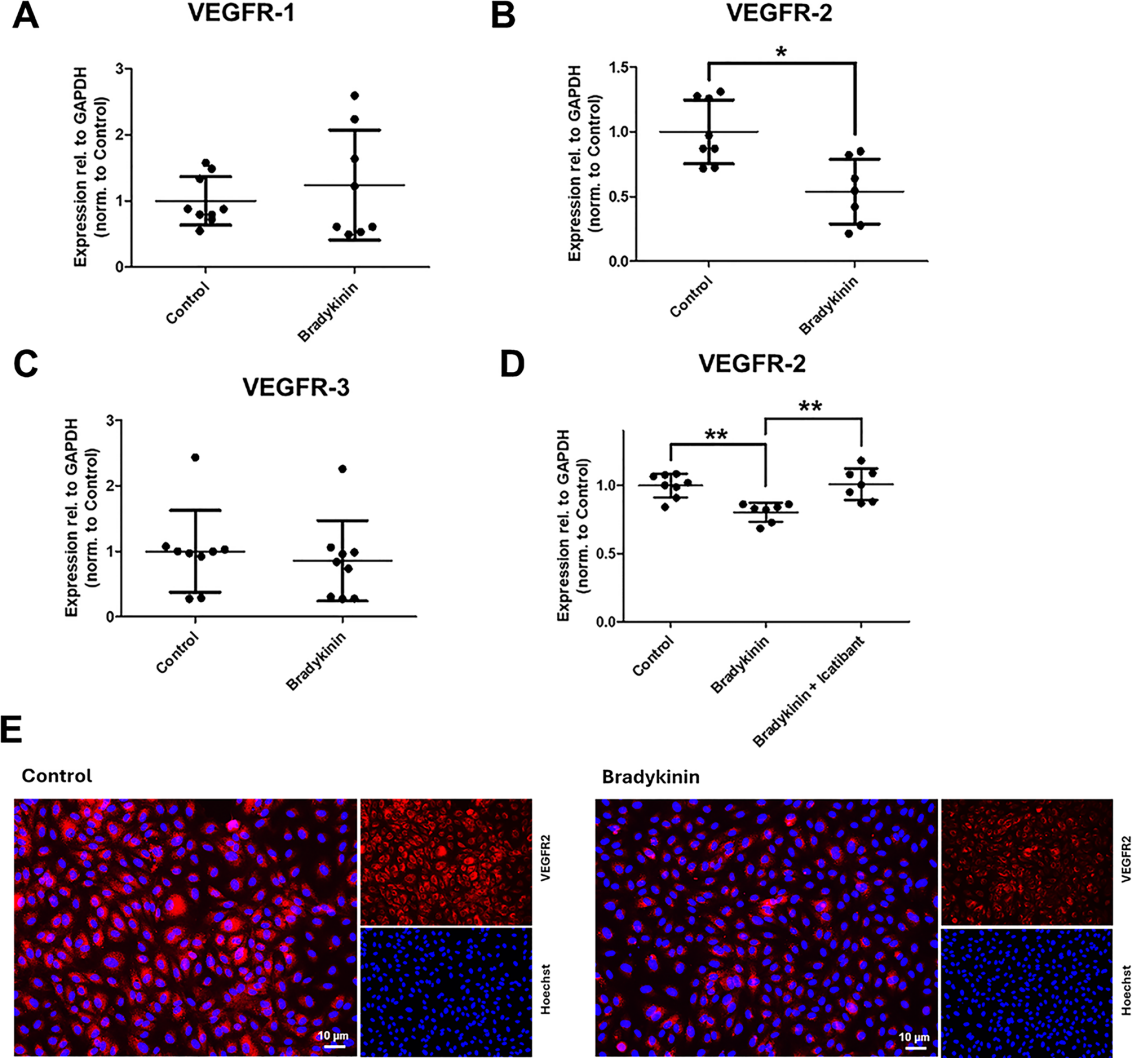
Bradykinin (100 µmol/l, 4 h) downregulates vascular endothelial growth factor receptor (VEGFR)-2. RT-PCR analysis showing expression of VEGFR-1 (**A**), VEGFR-2 (**B**), and VEGFR-3 (**C**). The bradykinin-induced downregulation of VEGFR-2 was reversed by the B2 receptor antagonist icatibant (10 nmol/l, 4 h; **D**). Expression levels were normalized to glyceraldehyde-3-phosphate dehydrogenase (GAPDH). Downregulation of VEGFR-2 was further confirmed by immunocytochemical staining (**E**; VEGFR-2 in red, nuclei in blue). The values are shown as scatter plots and the mean value with standard deviation is given. Each data point represents one filter. Significance was calculated using the two-tailed Mann-Whitney test (* = *p* < 0.05, ** = *p* < 0.01)

VEGF exerts its effects by binding to three different VEGF receptors. The influence of bradykinin on the three known receptors — VEGFR-1, VEGFR-2, and VEGFR-3 — was therefore investigated using RT-PCR and ICC (Fig. 2). Bradykinin treatment resulted in a downregulation of VEGFR-2, which could be counteracted by icatibant. The expression of VEGFR-1 and VEGFR-3 remained unaffected by bradykinin treatment.

### VEGFA has a slight impact on endothelial barrier function

**Fig. 3:**
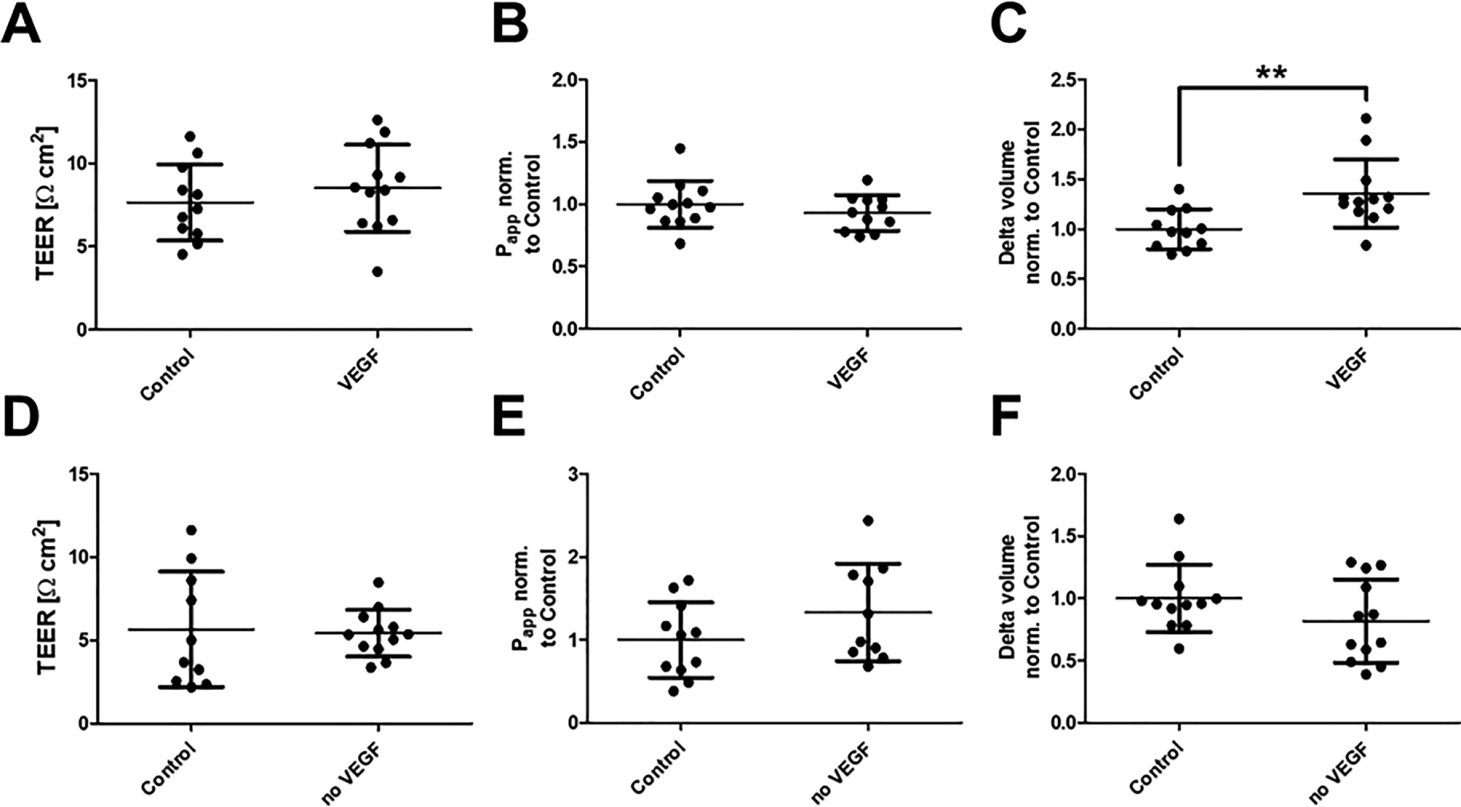
Influence of vascular endothelial growth factor (VEGF) on the endothelial barrier. Human umbilical vein endothelial cells were cultured on filter systems and exposed to different VEGF concentrations after three days for 4 h. While the control continued to be cultivated in a concentration of 0.5 ng/ml VEGF, one setup was exposed to an increased concentration of 100 ng/ml (VEGF). Another setup was placed on VEGF-free medium (no VEGF). The transendothelial electrical resistance (TEER, **A**/**D**) and the apparent permeability factor (P_app_, **B**/**E**) showed no significant change. In contrast, increased water permeability (**C**) was observed under higher VEGF concentration. No change was observed under VEGF-free medium (**F**). The values are shown as scatter plots and the mean value with standard deviation is given. Each data point represents one filter. Significance was calculated using the two-tailed Mann-Whitney test (* = *p* < 0.05)

Next, it was investigated whether bradykinin could lead to barrier dysfunction in this endothelial cell model by modulating VEGFA expression. Due to conflicting reports in the literature, the influence of VEGF on endothelial barrier function was first examined (Fig. 3). Since VEGF is already supplemented in the culture medium, two experimental setups were used: in one, the VEGF concentration (basal: 0.5 ng/ml) was increased to 100 ng/ml; in the other, VEGF-free medium was used.

Reducing VEGF levels had no effect on electrical resistance, apparent permeability, or transendothelial water flux. However, the addition of VEGF increased water permeability, while the other two parameters remained unchanged.

### VEGF has no impact on bradykinin-mediated barrier dysfunction

**Fig. 4:**
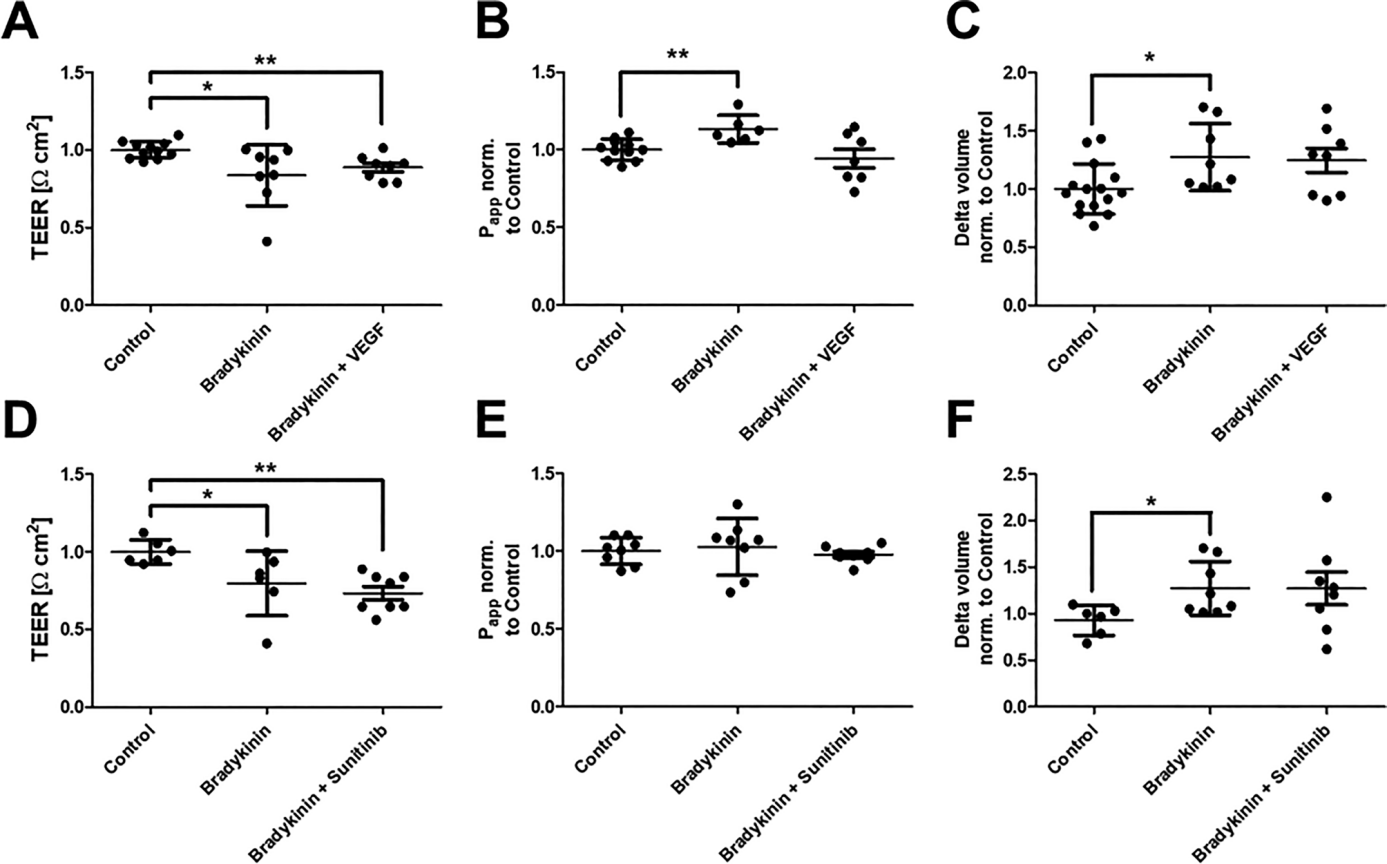
The vascular endothelial growth factor (VEGF) signaling pathway shows no modulation of bradykinin mediated barrier disruption. Bradykinin was applied to human umbilical vein endothelium for 4 h. In addition, 100 ng/ml vascular endothelial growth factor (VEGF) or 100 nM of the VEGF antagonist sunitinib was added. Measurements of transendothelial electrical resistance (TEER, **A**/**D**), apparent permeability (P_app_, **C**/**E**) and transendothelial water flux (**C**/**F**) consistently showed an increase in endothelial permeability to bradykinin. This was not significantly affected by VEGF or sunitinib. The values are shown as scattered plots, and the mean value with the standard deviation is given. Each data point represents one filter. Significances were calculated using the two-tailed Mann Whitney test in case of multiple testing a Bonferroni correction was additionally performed (* = *p* < 0.05, ** = *p* < 0.01)

In the final step, it was investigated whether VEGF influences bradykinin-induced barrier disruption. For this purpose, bradykinin was supplemented either with 100 ng/ml VEGF or with the VEGF inhibitor sunitinib (Fig. 4). Neither treatment significantly altered the effect of bradykinin on the endothelial barrier.

### Proliferation and migration of HUVECs is lower under bradykinin addition

**Fig. 5:**
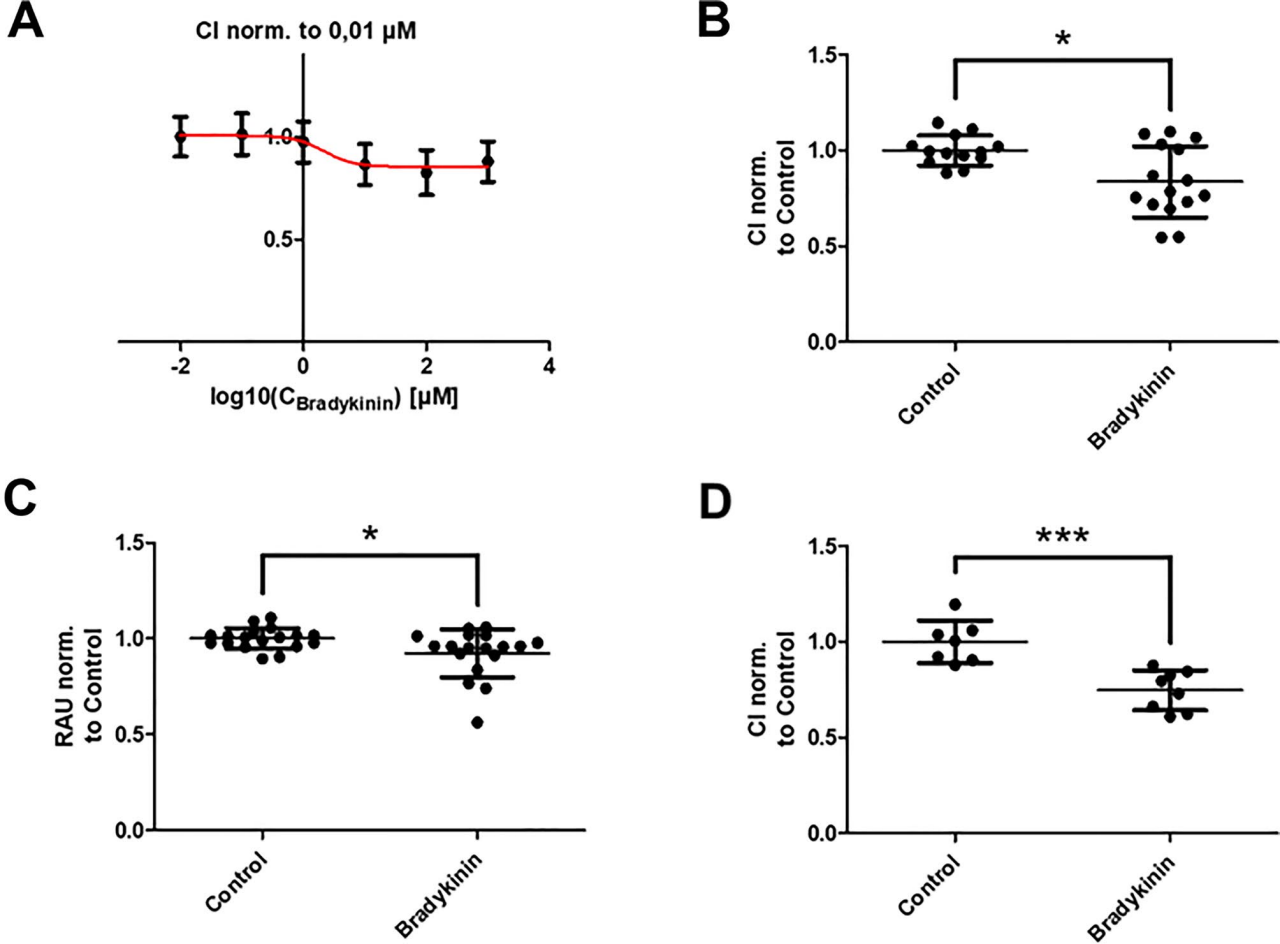
Bradykinin has a negative effect on proliferation and migration of human umbilical vein endothelial cells (HUVEC). A concentration-response curve (**A**) demonstrated a dose-dependent effect of bradykinin (4 h) in measurements obtained using real-time cell analysis (RTCA) technology. Electrical impedance is represented as the cell index (CI), and the slope of the cell index was monitored over time. This was confirmed in a second RTCA approach using 100 µmol/l bradykinin (**B**). Here CI was significantly reduced. Also, resazurin assay (**C**) showed a significant reduction in signal (RAU = relative arbitrary unit) after the addition of bradykinin, indicating a reduced cell number and therefore reduced proliferation. The cell invasion and migration (CIM) module of the RTCA technology was used to quantify HUVEC migration. Vascular endothelial growth factor was used as a chemoattractant (**D**). Again, bradykinin led to a significant reduction. The values are shown as scatter plots and the mean value with standard deviation is given. Each data point represents one filter. The concentration–response curve is presented as mean ± standard error of the mean (SEM) together with a fitted regression curve. Significance was calculated using the two-tailed Mann-Whitney test (* = *p* < 0.05, *** = *p* < 0.001)

Previous results showed that bradykinin affects the expression of VEGFA and its receptor; however, VEGFA had no impact on bradykinin-mediated barrier disruption. To investigate whether bradykinin induces a wound healing defect in line with the second-hit hypothesis, its effects on HUVEC proliferation and migration were examined first (Fig. 5). Proliferation was assessed colorimetrically using the resazurin assay and RTCA. A significant decrease in proliferation was observed following bradykinin treatment. Endothelial migration was determined using the CIM assay, which again showed a significant reduction upon bradykinin exposure. In contrast, the addition of VEGF significantly increased the proliferation rate (Supplementary Fig. 1).

### Scratch assays show impaired endothelial proliferation and migration under bradykinin

**Fig. 6:**
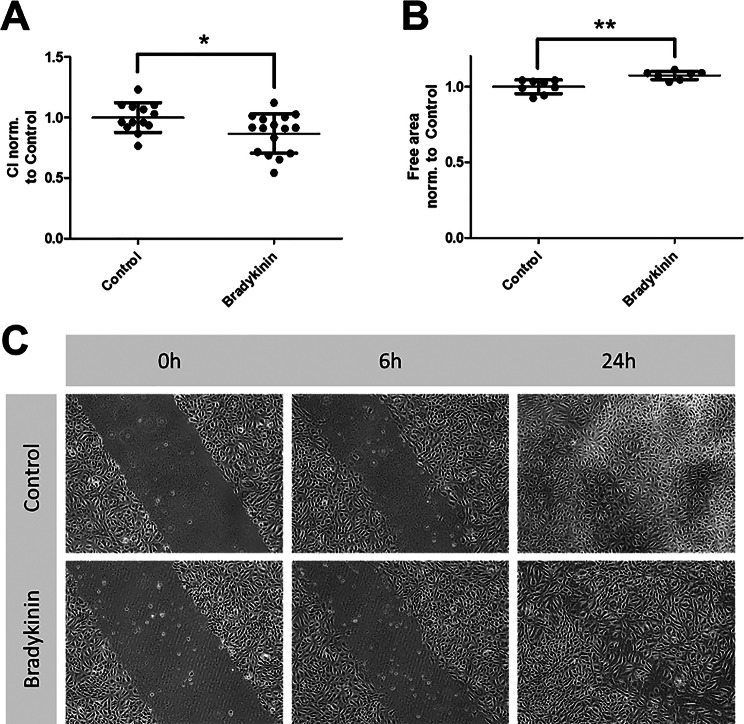
Endothelial wound healing is negatively influenced by bradykinin. To analyze the direct influence on wound healing, scratch assays were performed. Real-time cell analysis (RTCA, electrical capacity given as cell index = CI) technology (**A**) and microscopic examination (**B**) were used. In both cases, bradykinin showed delayed endothelial wound healing. (**C**) shows representative images of the performed scratch assay during incubation with 100 µmol/l bradykinin compared to control. The first image was taken immediately after scratching the confluent cell layer, after that images were taken at 6 h for analysis. The pictures shown at 24 h show a nearly completed closure of the scratch. The values are shown as scatter plots and the mean value with standard deviation is given. Each data point represents one filter. Significance was calculated using the two-tailed Mann-Whitney test (* = *p* < 0.05, ** = *p* < 0.01)

Endothelial wound healing is primarily dependent on the proliferation and migration of endothelial cells. The scratch assay provides indirect evidence of this (Fig. 6). The addition of bradykinin significantly reduced both proliferation and migration of the endothelium, while VEGF treatment improved these processes, as expected (Supplementary Figure 1). These findings suggest that bradykinin may impair endothelial wound healing.

### VEGF antagonizes bradykinin mediated impaired wound healing capacity

**Fig. 7:**
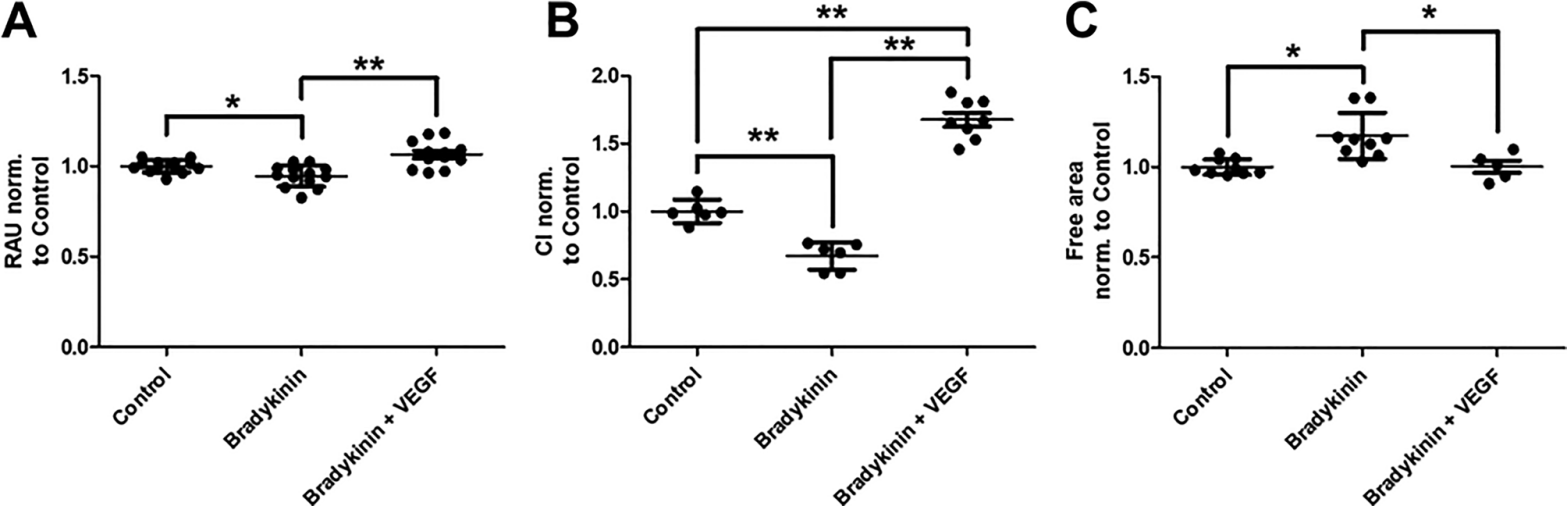
Vascular endothelial growth factor (VEGF) antagonizes the negative influence of bradykinin on endothelial wound healing. To demonstrate that the impaired wound healing was caused by the downregulation of VEGFA and its receptor VEGFR-2, VEGF was also added to the cells. In the resazurin assay (RAU = relative arbitrary unit) bradykinin caused a reduction that was completely reversed by VEGF (**A**). Capacity measurements using real-time cell analysis (RTCA, electrical capacity is given as cell index = CI) confirmed this (**B**). Finally, scratch assays were performed in which the free area after mechanical damage was analyzed over time (**C**). Again, a bradykinin mediated delay in wound healing was observed, which could be inhibited by VEGF. The values are shown as scattered plots, and the mean value with the standard deviation is given. Each data point represents one filter. Significances were calculated using the two-tailed Mann Whitney test in case of multiple testing a Bonferroni correction was additionally performed (* = *p* < 0.05, ** = *p* < 0.01)

Finally, a potential link was explored between the reduced VEGFA expression observed earlier and the impaired endothelial wound healing induced by bradykinin. To test this, it was examined whether increased VEGFA expression could counteract the effects of bradykinin (Fig. 7). Both the resazurin and RTCA assays demonstrated that VEGF clearly reversed the bradykinin-mediated reduction in proliferative capacity. In the scratch assay, the bradykinin-induced impairment in wound closure was also completely antagonized by the addition of VEGF. When directly compared, VEGF alone promoted wound closure, whereas co-treatment with VEGF and bradykinin resulted in less improvement (Supplementary Fig. 2).

## Discussion

Bradykinin reduces blood flow by inducing vasodilation and enhances leukocyte migration into tissues by increasing endothelial permeability [22]. However, at excessive concentrations, the resulting increase in permeability leads to the extravasation of water and solutes from the intravascular space into the interstitium. This causes the recurrent onset of edema, which is characteristic of HAE. According to the second hit hypothesis, a genetic defect or baseline barrier disorder creates a predisposition to damage, but a second trigger is required for the clinical manifestation of symptoms [23–25]. We hypothesized that elevated bradykinin levels in HAE patients may delay endothelial wound healing and thereby promote edema formation. Wound healing is primarily dependent on two cellular processes: proliferation and migration [26].

We observed a significant reduction in both VEGFA and VEGFR-2 expression. Since VEGFA primarily signals via VEGFR-2, these findings are consistent [27]. In contrast, other studies have reported increased VEGF expression following bradykinin treatment [16]. However, these studies employed different endothelial cell models, suggesting tissue-specific effects. Moreover, the isoform of VEGF is a crucial variable. For example, VEGFC plays an essential role in the proliferation and differentiation of lymphatic endothelial cells [28], whereas VEGFA is more relevant for vascular endothelial function—consistent with our cell model [29]. The previously mentioned study also used a lymphatic endothelial model, which may explain the observed discrepancies. A clinical study examining blood samples from HAE patients revealed elevated VEGFA and VEGFC levels during remission compared to healthy controls. Additionally, increased VEGFA and VEGFC levels were associated with higher attack frequency [30]. Another study, however, found no change in VEGF levels during acute HAE attacks [31]. While this initially appears contradictory, several factors should be considered: These studies assessed only VEGF isoforms—not their receptors—which are equally critical for signaling. Furthermore, they analyzed systemic blood samples, which may not accurately reflect localized reactions such as swelling attacks. Thus, such local effects could be easily missed. Nevertheless, the data collectively suggest a potential link between bradykinin and VEGF signaling.

Regarding the impact of bradykinin on endothelial proliferation, another study investigated this in the context of cervical cancer [32]. In this setting, bradykinin was reported to have a positive effect on angiogenesis, using a concentration of 10 µmol/l and a tube formation assay. It should be noted, however, that the HUVEC in that study were cultured under substantially different conditions (3D culture in Matrigel). Moreover, these experiments did not assess cell proliferation per se, but rather the capacity for tube formation, which reflects angiogenic potential rather than endothelial cell growth. In contrast, we focused on proliferation analyses, as these are more relevant for the pathophysiology of angioedema, where angiogenesis plays a minimal role. Another factor may be the lower concentration of bradykinin used in our experiments, since our data also demonstrated a dose-dependent effect. In our experiments, we used a bradykinin concentration of 100 µmol/l. This concentration was selected based on several considerations. First, the concentration–response curve demonstrated a stable proliferative effect at this level. Second, previous studies in HUVEC using the same concentration revealed measurable effects on the endothelial barrier, which could be antagonized by the B2 receptor antagonist icatibant, indicating a specific, reproducible response [20]. Third, icatibant was also protective against the bradykinin-mediated downregulation of VEGFA and VEGFR-2 in our analyses. Notably, even a relatively low concentration of icatibant was sufficient to block the effect of bradykinin. This can be explained by several pharmacodynamic properties of icatibant: it has a very high affinity for the B2 receptor, a substantially lower dissociation rate, and is proteolytically stable [33–35]. Together, these features allow icatibant to maintain predominant receptor occupancy over the four-hour observation period used in our experiments, whereas bradykinin is comparatively short-lived. Consequently, even at a much lower nominal concentration, icatibant can effectively outcompete bradykinin and prevent receptor activation, supporting the specificity and reproducibility of the observed response. Lower concentrations of bradykinin might have yielded weaker or less consistent effects, as our data show a clear dose dependence. Importantly, local bradykinin concentrations at the site of edema formation may far exceed serum levels, justifying the use of higher concentrations in vitro to mimic physiologically relevant local exposure. Finally, other studies employing lower concentrations or different experimental readouts (e.g., tube formation assays for angiogenesis in tumor models) cannot be directly compared, as proliferation analyses are more relevant to the pathophysiology of angioedema.

Following the observed downregulation of VEGFA and VEGFR-2, we investigated whether VEGFA contributes to bradykinin-induced endothelial barrier disruption. Because VEGFA is already supplemented in standard endothelial culture media, we employed three experimental conditions: (1) a basal concentration of 0.5 ng/ml VEGFA, (2) an increased concentration of 100 ng/ml, and (3) no VEGFA. Reducing the VEGFA concentration had no effect on endothelial barrier function. Similarly, increased VEGFA had no impact on TEER or dextran (70 kDa) permeability. However, it significantly increased transendothelial water flux. The first two methods are classic markers of endothelial integrity and were not modulated in HUVECs—consistent with previous findings [19]. Again, the literature reveals strong tissue specificity in VEGF responses [36, 37]. For instance, VEGF reduces barrier function in some endothelial cell types but not in others, such as HUVECs. Additionally, the specific VEGF isoform likely plays an important role. The D₂O dilution method directly quantifies endothelial water permeability [20], and it demonstrated that VEGFA significantly increases water flux, suggesting that it does affect endothelial function in HUVECs to some extent. However, its contribution to bradykinin-mediated barrier disruption appears limited. On one hand, bradykinin reduces VEGFA expression; on the other, it clearly disrupts endothelial integrity—beyond simply increasing water permeability [20]. To investigate this further, bradykinin was administered along with either 100 ng/ml VEGFA or sunitinib. Sunitinib is a multi-targeted receptor tyrosine kinase inhibitor. Receptor tyrosine kinases that are inhibited by sunitinib are among others VEGFR-1 and − 2, but also platelet-derived growth factor receptors, stem cell factor receptor (KIT), FMS-like tyrosine kinase-3, glial cell-line derived neurotrophic factor receptor and the receptor of macrophage-colony stimulating factor [38]. The known negative effect of bradykinin on the endothelial barrier was again observed. However, neither the addition of VEGFA nor its blockade altered this effect, suggesting that bradykinin-induced barrier disruption is independent of VEGF signaling. We then shifted focus to the role of VEGF in endothelial wound healing, which depends largely on proliferation and migration [39]. Consistent with this, our data confirmed a positive effect of VEGFA on HUVEC wound healing. In contrast, the influence of bradykinin on these processes has been less well studied. Proliferation and migration were therefore examined in more detail. Previous studies have linked bradykinin to changes in these processes [40–42].

The resazurin assay detects mitochondrial reduction of resazurin to fluorescent resorufin, allowing snapshot measurements of viable cells. RTCA continuously monitors cell adherence and proliferation through impedance measurements via gold electrodes, enabling real-time tracking of proliferation. Both methods revealed in our study that bradykinin reduces endothelial proliferation. Migration was analyzed using the CIM assay, a 3D approach similar to a Boyden chamber [43]. VEGF was used as a chemoattractant to assess VEGF-dependent migration. As expected, migration was reduced in bradykinin-treated cells, consistent with the earlier observed downregulation of VEGFR-2, which likely impairs chemoattractant sensing. To evaluate wound healing—an interplay of proliferation and migration—we employed scratch assays. Although RTCA-based scratch assays are not yet standardized, our CI data were consistent and reproducible. Thus, RTCA complements conventional approaches, providing an objective method for wound healing assessment. Again, bradykinin impaired scratch closure, confirming a negative effect on wound healing. Finally, we assessed whether the bradykinin-induced impairment of wound healing was mediated by VEGF. Co-administration of VEGFA with bradykinin completely reversed the wound healing deficit. However, VEGF alone promoted endothelial proliferation and wound closure compared to control conditions. When VEGF was co-administered with bradykinin, this effect was less pronounced and no longer significant, suggesting that bradykinin may interfere with VEGF-mediated wound healing. One possible explanation could be the bradykinin-induced downregulation of VEGFR-2, which may reduce cellular responsiveness to VEGF.

## Conclusion

Our study demonstrates that bradykinin negatively affects endothelial proliferation and migration in HUVEC, while its barrier-disruptive effect is independent of VEGF signaling. Bradykinin reduces the expression of VEGFA and VEGFR-2, and the impaired proliferation and migration can be attributed to VEGF downregulation. These findings suggest that bradykinin can contribute to endothelial dysfunction in angioedema, with VEGF-dependent pathways mediating effects on proliferation and migration. This highlights a mechanistic link between elevated local bradykinin levels and impaired endothelial repair, providing insights into the pathophysiology of HAE and other edema-related conditions in context of the second-hit hypothesis.

## Supplementary Information

Below is the link to the electronic supplementary material.

Supplementary Material 1

Supplementary Material 2

Supplementary Material 3

## Data Availability

The data that support the findings of this study are available from the corresponding author upon reasonable request.

## Abbreviations

C1INH: C1 esterase inhibitor
HAE: Hereditary angioedema
HUVECs: Human umbilical vein endothelial cells
ICC: Immunocytochemistry
P_app_: Apparent permeability coefficient
PLGF: Placenta growth factor
RTCA: Real time cell analysis
RT-PCR: Real time - Polymerase chain reaction
TEER: Transendothelial electrical resitance
VEGF: Vascular endothelial growth factor
VEGFR: Vascular endothelial growth factor receptor
WB: Western Blot
