# Supplementary figures and images for: Bradykinin reduces wound healing in human umbilical vein endothelial cells via downregulation of vascular endothelial growth factor A
Source: J Inflamm (Lond). 2026 Jan 10;23:2. doi: 10.1186/s12950-026-00485-x (PMC12849120; doi:10.1186/s12950-026-00485-x)

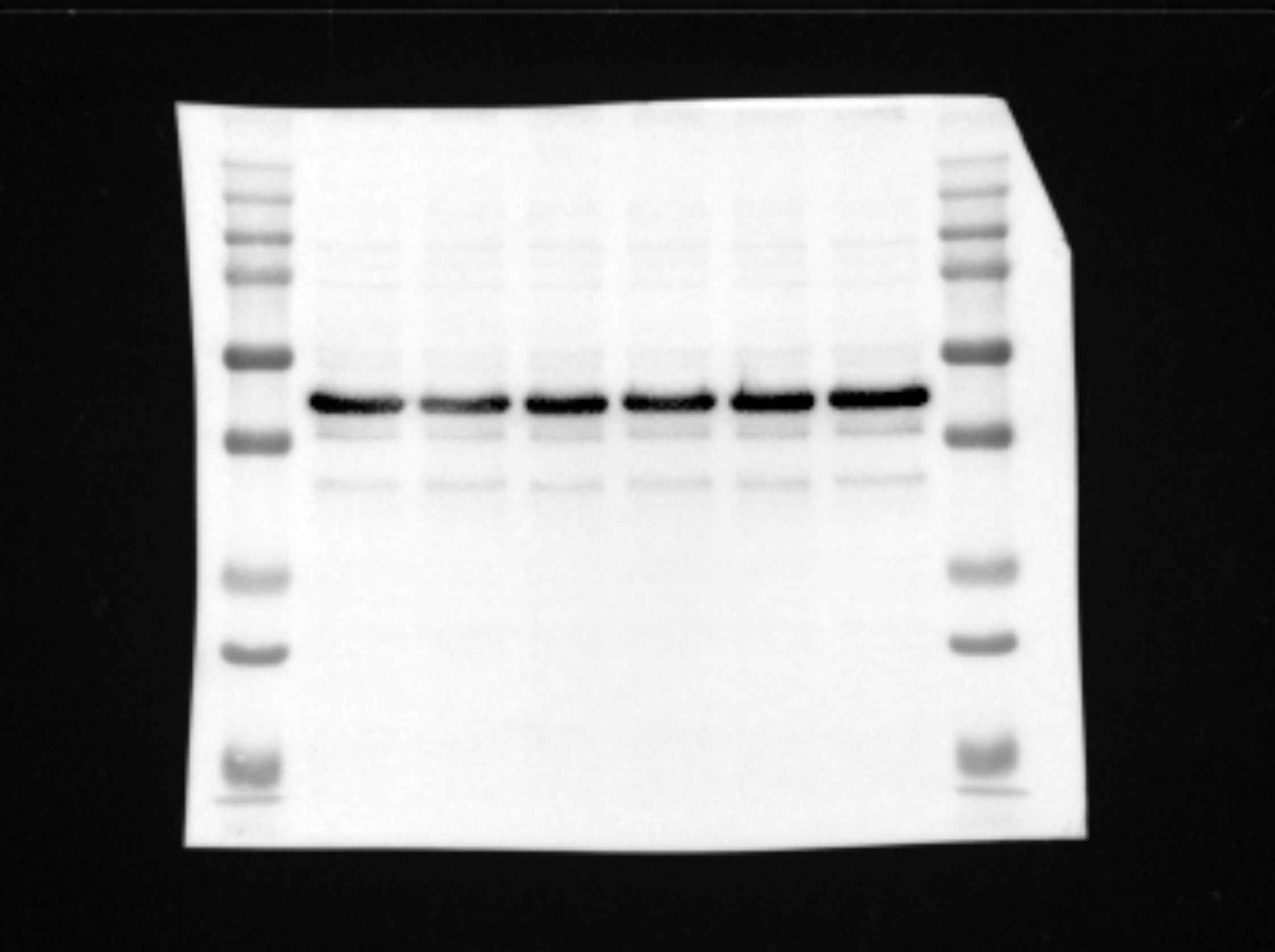

Supplement: Supplementary file 1 — Supplementary Material 1 [file 12950_2026_485_MOESM1_ESM.jpg]

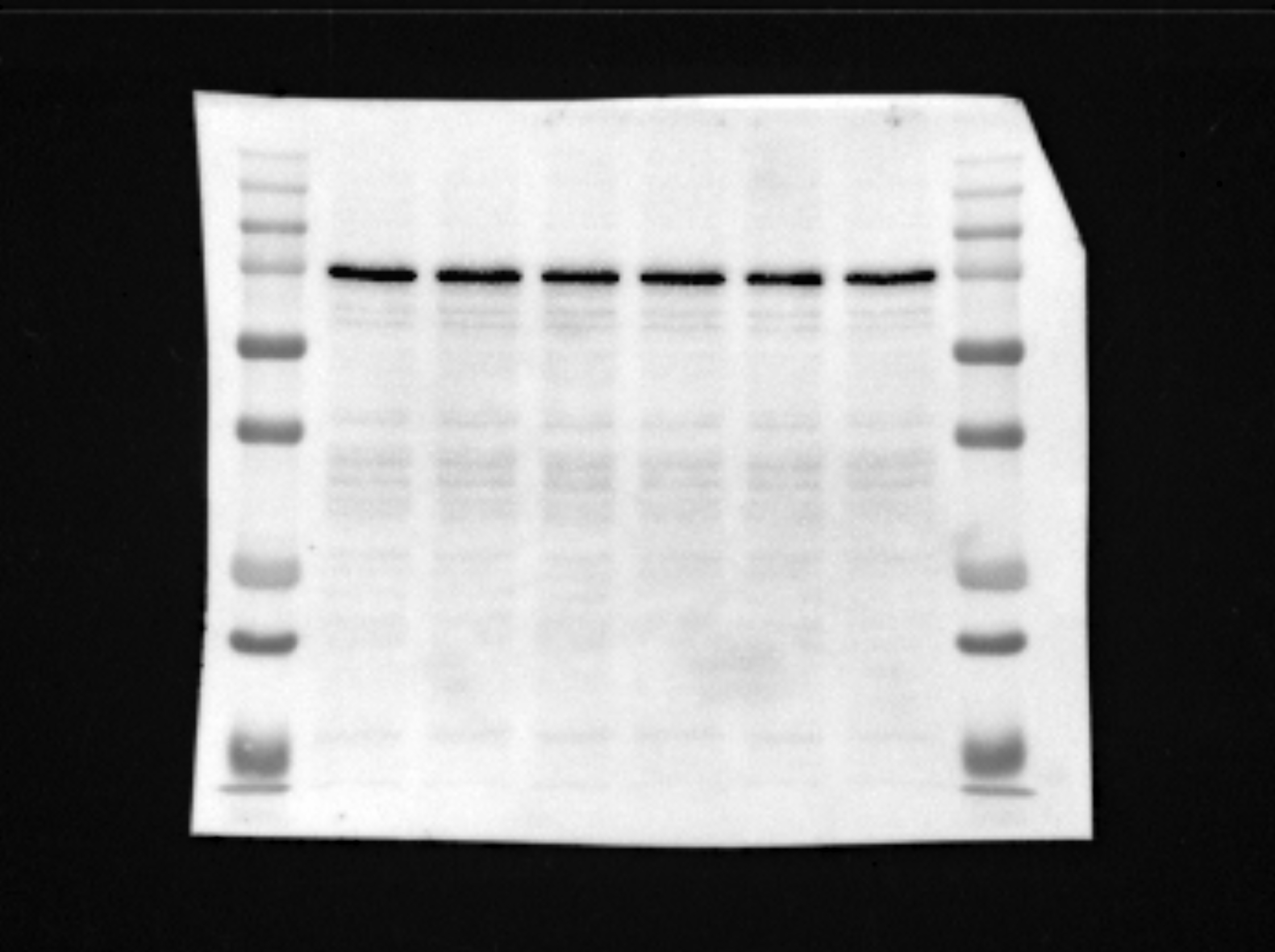

Supplement: Supplementary file 2 — Supplementary Material 2 [file 12950_2026_485_MOESM2_ESM.jpg]
