# Supplementary material for: Bradykinin reduces wound healing in human umbilical vein endothelial cells via downregulation of vascular endothelial growth factor A
Source: J Inflamm (Lond). 2026 Jan 10;23:2. doi: 10.1186/s12950-026-00485-x (PMC12849120; doi:10.1186/s12950-026-00485-x)
Supplement: Supplementary file 3 — Supplementary Material 3 [file 12950_2026_485_MOESM3_ESM.docx]

Supplementary figures

*
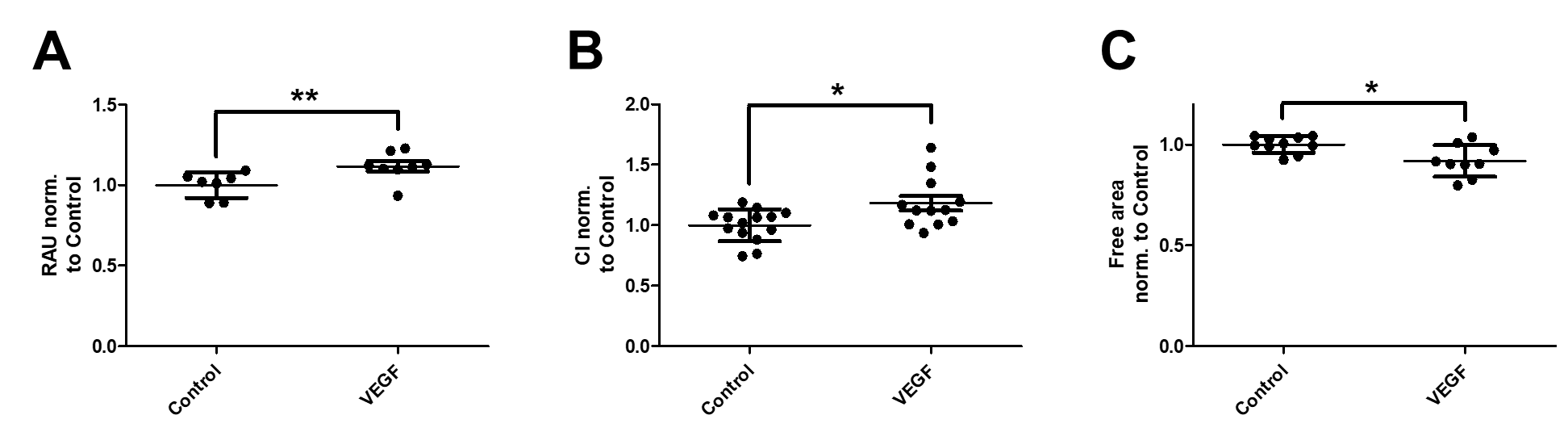
Supplementary figure 1*

Supplementary Figure 1: Vascular endothelial growth factor (VEGF) promotes endothelial wound healing. Using the resazurin assay (A, RAU = relative arbitrary unit) and real-time cell analysis (B, CI = cell index), VEGF was shown to promote endothelial proliferation. This was accompanied by an improvement in wound healing in the scratch assay (C). A significant reduction in free area was observed over time following mechanical injury. The values are shown as scatter plots and the mean value with standard deviation is also given. Each data point represents one filter. Significance was calculated using the two-tailed Mann-Whitney test (* = p < 0.05, ** = p < 0,01).

*
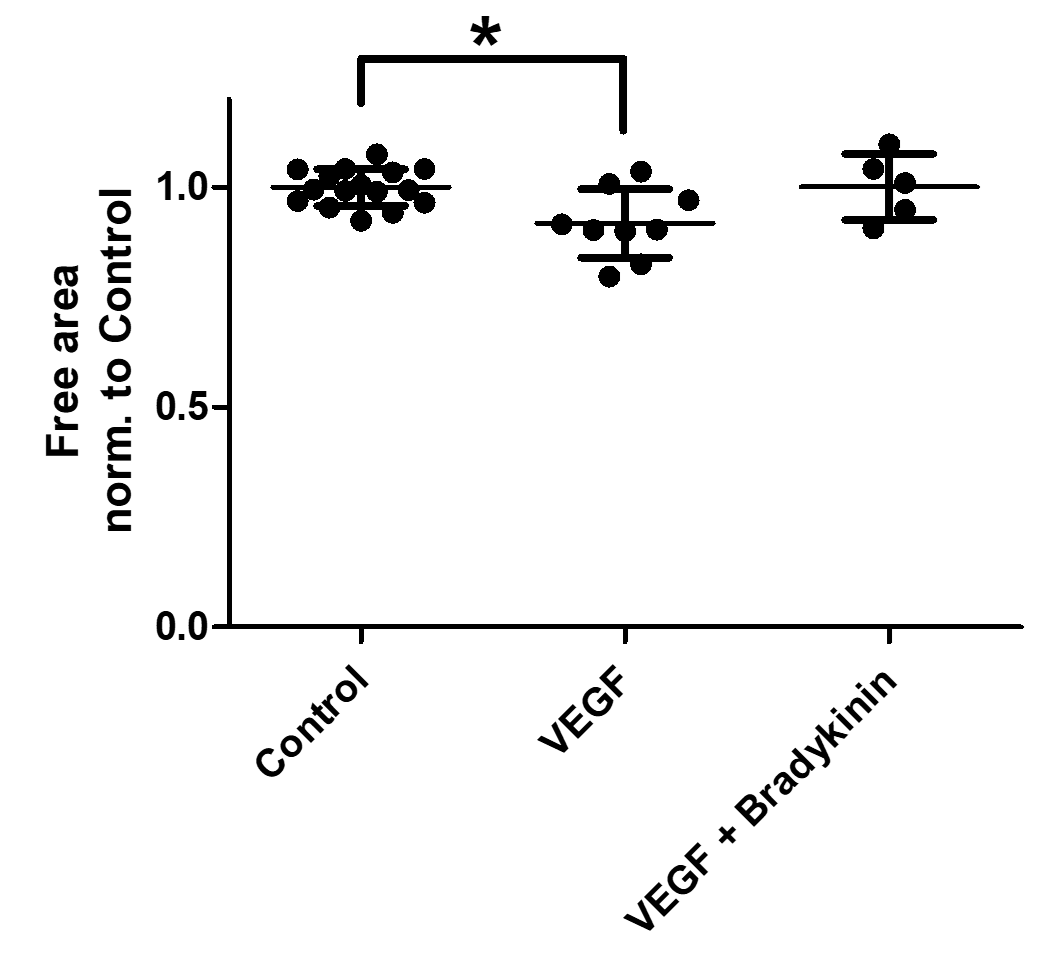
Supplementary figure 2*

Supplementary Figure 2: Comparison between vascular endothelial growth factor (VEGF) alone and in addition with bradykinin on endothelial proliferation. Scratch assays were performed to assess wound closure after24 h. Treatment with VEGFA alone (100 ng/ml) promoted wound healing, as indicated by a reduction in free area. Co-treatment with bradykinin (100 µM) attenuated this VEGF-induced effect. Data are shown as scatter plots with mean ± SD. Each data point represents one filter. Significance was calculated using a two-tailed Mann-Whitney test (** p < 0.01).
